# Voltage-Dependent Emission Varying from Blue to Orange–Red from a Nondoped Organic Light-Emitting Diode with a Single Emitter

**DOI:** 10.3390/nano12142333

**Published:** 2022-07-07

**Authors:** Mingxue Yang, Tian-Xiang Zhao, Si-Chao Ji, Xiao-Dong Tao, Xu-Lin Chen, Lingyi Meng, Dong Liang, Can-Zhong Lu

**Affiliations:** 1 CAS Key Laboratory of Design and Assembly of Functional Nanostructures, and Fujian Provincial Key Laboratory of Nanomaterials, Fujian Institute of Research on the Structure of Matter, Chinese Academy of Sciences, Fuzhou 350002, China; xmyangmingxue@fjirsm.ac.cn (M.Y.); xmzhaotianxiang@fjirsm.ac.cn (T.-X.Z.); xmjisichao@fjirsm.ac.cn (S.-C.J.); taoxiaodong17@mails.ucas.edu.cn (X.-D.T.); xlchem@fjirsm.ac.cn (X.-L.C.); lymeng@fjirsm.ac.cn (L.M.); dl@fjirsm.ac.cn (D.L.); 2 Xiamen Institute of Rare Earth Materials, Fujian Institute of Research on the Structure of Matter, Chinese Academy of Sciences, Xiamen 361021, China; 3 University of Chinese Academy of Sciences, Beijing 100049, China

**Keywords:** electrochromic, emission color, excimer, organic light-emitting diodes, white OLEDs

## Abstract

Organic light-emitting diodes (OLEDs) with tunable emission colors, especially white OLEDs, have rarely been observed with a single emitter in a single emissive layer. In this paper, we report a new compound featuring a D–A–D structure, 9,9′-(pyrimidine-2,5-diylbis(2,1-phenylene))bis(3,6-di-tert-butyl-9*H*-carbazole) (**PDPC**). A nondoped OLED using this compound as a single emitter exhibits unique voltage-dependent dual emission. The emission colors range from blue to orange–red with an increase in voltage, during which white electroluminescence with a *Commission Internationale De L’Eclairage* (CIE) coordinate of (0.35, 0.29) and a color render index (CRI) value of 93 was observed. A comparative study revealed that the dual emission simultaneously originates from the monomers and excimers of the emitter. This study provides insight into understanding the multimer-excited mechanism and developing novel color-tunable OLEDs.

## 1. Introduction

Organic light-emitting diodes (OLEDs) have many applications in displays and lighting due to their high efficiency, flexibility, and ultrathin thickness [1,2,3]. In particular, the application of color-tunable OLEDs is of wide-ranging demand in the fields of decoration, smart lighting, cultivating vegetables, wearable sensing equipment, etc. [4,5,6,7,8]. In general, three types of strategies have been proposed for color-tunable devices [9]: The first strategy involves the combination of two or three materials with different emissions, including deploying tandem structures that contain sub-OLED arrays and share one electrode [4,10,11,12]. The complicated structure and fabrication cost of this device, however, discourage this option. One alternative is to deposit multiple emitters in a single cell, by either layer-by-layer doping or co-doping [6,13,14,15]. Despite their facilitated fabrication, the device obtained from the latter method suffers from the asynchronous color aging of the emitters. The device with a single emitter can be a solution to this deficit.

Concerning single emitters, dual-emission capability is their fundamental requirement, meaning the compound can emit both emissions in different excited states. In addition, for better discernibility of color change, the variation trend of the emission intensities of the two peaks should be in distinctive pace with the alteration in the voltage. in addition to the emission of monomers, the emission of multimers is one of the methods for generating a lower-energy emission. Mazzeo et al. reported an A–D–A structure with double dimesitylboyl groups connected by a centering terthiophene [16]. This compound exhibited an emission in the blue region with an additional red-shifted peak, while the author attributed the latter peak to the formation of dimers. However, the maximum external quantum yield (EQE) was 0.35%, and the turn-on voltage was around 9 V.

In this paper, we present a new compound, 9,9′-(pyrimidine-2,5-diylbis(2,1-phenylene))bis(3,6-di-tert-butyl-9*H*-carbazole) (**PDPC**), featuring a donor-acceptor-donor (D–A–D) structure. Employing this compound as a single emitter, a single OLED cell with a single undoped emissive layer exhibited voltage-dependent dual emission. As the voltage increased, the emission color ranged from blue to orange–red. A comparison between the nondoped and doped OLEDs revealed that the red peak resulted from the multimerization of the emitter molecules. The maximum EQE was 0.81%, and the turn-on voltage was 5.19 V. The wide color range observed revealed that the electroluminescent (EL) emission varied from blue (CIE_1931_ = (0.17, 0.20)) to orangish red (CIE_1931_ = (0.50, 0.39)). In addition, white electroluminescence with a CIE coordinate of (0.35, 0.29) and a CRI value of 93 was observed.

## 2. Materials and Methods

### 2.1. General Information

All reaction experiments were performed under a N_2_ atmosphere using standard Schlenk techniques, unless specified. The organic materials investigated in this study were synthesized by the procedures described below, in which the starting materials (all solvents and reagents) were purchased from commercial sources and were used as received without further purification. (For detail: Argon: Linde plc, Dublin, Ireland; 2,5-dibromopyrimidine, palladium(II) diacetate, 2-fluorophenylboronic acid, 3,6-di-tertbutyl-9*H*-carbazole: Bide Pharmatech Ltd, Shanghai, China; K_3_PO_4_·3H_2_O, silica gel, Cs_2_CO_3_, tetrahydrofuran: China National Pharmaceutical Group Co., Ltd. (Sinopharm), Beijing, China; glycol, NaCl, petroleum ether, ethyl acetate, dichloromethane, dimethylformamide (DMF), deuterated dimethyl sulfoxide (DMSO-*d*_6_): Shanghai Titan Scientific Co., Ltd., Shanghai, China.)

### 2.2. Preparation of 2,5-Bis(2-fluorophenyl)pyrimidine

Under an argon atmosphere, 2.38 g (10 mmol) of 2,5-dibromopyrimidine, 0.112 g (5 wt%) of palladium(II) diacetate (preheated in an oven at 80 °C for 3 h), and 7.99 g (3 equiv., 30 mmol) of K_3_PO_4_·3H_2_O (dried in an oven overnight at 150 °C) were mixed with 15 mL of glycol in a 100 mL Schlenk flask, and the solution was stirred for 10 min. Then, 4.20 g (3 equiv., 30 mmol) of 2-fluorophenylboronic acid was added into the solution. Following this, 3 purges and-refills of argon were performed. The solution turned into a yellowish-orange color. The mixture was heated at 80 °C for 24 h. After the reaction cooled to room temperature, the mixture was poured into a beaker with 150 mL of water, and the precipitate was obtained by vacuum filtration. The final product was purified by column chromatography filled with silica gel. The eluent was adopted by gradually increasing the content of ethyl acetate in petroleum ether, until ca. 10%. The half-substituted byproduct was separated, and a reaction similar to that one mentioned above was stoichiometrically adopted to obtain the fully substituted product. Scheme S1 diagramed the procedure. Total yield: 1.04 g, 38.8%. ^1^H NMR (400 MHz, DMSO-*d*_6_) δ 9.18 (d, *J* = 1.5 Hz, 2H), 8.10 (td, *J* = 7.8, 1.9 Hz, 1H), 7.78 (td, *J* = 7.8, 1.8 Hz, 1H), 7.63–7.53 (m, 2H), 7.47–7.36 (m, 4H). ^13^C NMR (101 MHz, DMSO-*d*_6_) δ 161.86, 161.32 (d, *J* = 4.7 Hz), 160.64, 159.33, 158.18, and 156.84 (d, *J* = 3.8 Hz), 132.51 (d, *J* = 8.6 Hz), 131.77, 131.36 (d, *J* = 8.3 Hz), 130.82 (d, *J* = 2.8 Hz), 126.91, 125.71 (d, *J* = 9.3 Hz), 125.46 (d, *J* = 3.6 Hz), 124.63 (d, *J* = 3.7 Hz), 121.72 (d, *J* = 13.4 Hz), 116.67 (dd, *J* = 57.2, 22.0 Hz). The original graph for 1H and 13C spectra can be found in Figure S1 and S2.Element analysis: calcd. for C_16_H_10_F_2_N_2_: C 71.64, H 3.76, and N 10.44; found: C 71.60, H 3.81, and N 10.43. 

### 2.3. Preparation of 9,9′-(Pyrimidine-2,5-diylbis(2,1-phenylene))bis(3,6-di-tert-butyl-9H-carbazole) (***PDPC***)

Before 0.537 g (2 mmol) of 2,5-bis(2-fluorophenyl)pyrimidine was added, 1.34 g (4.8 mmol, 2.4 equiv.) of3,6-di-tertbutyl-9*H*-carbazole, 1.96 g (6 mmol, 3 equiv.) of Cs_2_CO_3,_ and 10 mL of DMF were stirred for 30 min in a 100 mL Schlenk flask under an Ar atmosphere. The solution was refluxed for 16 h after all the reactants mingled. The color of the solution turned from brown to yellow after heating. The cooled mixture was poured into a beaker with 150 mL of water, and the precipitate was obtained by vacuum filtration. The final product was collected through the recrystallization of the mixed solution of dichloromethane and ethyl acetate. Scheme S2 diagramed the procedure. Total yield: 1.25 g, 79.4%. ^1^H NMR (500 MHz, chloroform-*d*) δ 8.03 (dd, *J* = 12.8, 1.9 Hz, 4H), 7.97 (s, 2H), 7.72 (dd, *J* = 7.8, 1.6 Hz, 1H), 7.58–7.50 (m, 3H), 7.50–7.40 (m, 3H), 7.24 (ddd, *J* = 8.3, 6.1, 1.9 Hz, 4H), 7.12 (dd, *J* = 7.8, 1.7 Hz, 1H), 6.92 (d, *J* = 8.6 Hz, 2H), 6.80 (d, *J* = 8.5 Hz, 2H), 1.41 (s, 18H), 1.39 (s, 18H). ^13^C NMR (101 MHz, chloroform-*d*) δ 163.57, 155.21, 142.96, 142.30, and 139.46 (d, *J* = 33.0 Hz), 136.43, 135.92 (d, *J* = 22.9 Hz), 133.70, 131.79, and 131.15 (d, *J* = 16.7 Hz), 130.50, 130.06, 129.72, 129.16, 128.85, 127.87, 123.81, and 123.51 (d, *J* = 9.0 Hz), 123.19, 116.56, 115.89, and 109.02 (d, *J* = 14.0 Hz), 34.78, 32.10 (d, *J* = 6.6 Hz). The original graph for 1H and 13C spectra can be found in Figure S3 and S4. Element analysis: calcd. for C_56_H_58_N_4_: C 85.45, H 7.43, and N 7.12; found: C 85.39; H 7.43, and N 7.09.

### 2.4. Characterization

^1^H NMR and the spectra were recorded with a Bruker Avance III 400 MHz NMR spectrometer (Bruker Co., Billerica, MA, USA) with DMSO-*d*_6_ as a solvent. The chemical shifts are given in parts per million with reference to tetramethylsilane (TMS, *δ* = 0 ppm). The peak multiplicities are reported with the notation s (singlet), d (double), t (triplet), q (quartet), and m (multiplet). The elemental analyses (C, H, and N) were implemented with an Elementary Vario EL III elemental analyzer (Elementar Analysensysteme GmbH, Langenselbold, Germany). The thermogravimetric analysis (TGA) of the samples was performed with a METTLER TOLEDO TGA/DSC 1 STARe System (Mettler Toledo International Inc., Columbus, Oh, USA) with a heating rate of 10 °C/min under nitrogen. The UV–visible absorption spectra were determined with a PerkinElmer Lambda 365 spectrophotometer (PerkinElmer, Waltham, MA, USA) under ambient conditions. The steady-state photoluminescence and phosphorescence spectra at 300 K were measured using a Hitachi F-7000 (Hitachi Limited, Tokyo, Japan). The photoluminescence spectra at 77 K and time-resolved photoluminescent-decay experiments (lifetime) were performed with an Edinburgh Analytical instrument FLS980 (Edinburgh Analytical Instrument Limited, Livingston, UK), equipped with a xenon arc (450 W) and pulsed flash lamps. The delayed emission spectra in solution were recorded with an Ocean Optics MX2500+ (Ocean Insights, Inc., Orlando, FL, USA), equipped with a xenon arc lamp (450 W). The cyclic voltammetry (CV) analysis was performed on a CHI840D Electrochemical Analyzer (CH Instruments, Inc., Bee Cave, TX, USA), in which dichloromethane and 0.1 mol/L of tetrabutylammonium hexafluorophosphate were adopted as the solvent and electrolyte, respectively. The work, counter, and reference electrodes were glassy carbon, platinum wire, and Ag/AgNO_3_, respectively. The solution was pretreated with five-minute degassing by N_2_ before the CV test. The highest occupied molecular orbital (HOMO) was estimated as follows: HOMO = −[*E*_ox_ − *E*_Fc/Fc+_ + 4.8] eV.

### 2.5. Device Fabrication and Characterization

Glass substrates precoated with 120 nm of indium tin oxide (ITO) with a sheet resistance of 15 Ω per square were successively cleaned in an ultrasonic bath of deionized water, acetone, and isopropanol for 15 min. Then, the ITO glass substrates were dried with Ar_2_ steam and treated with UV-ozone for 15 min. The organic materials for the other functional layers were spin-coated onto the ITO-coated substrates at a rate of 1 Å s^−1^ under a high vacuum level (<2 × 10^−5^ Pa) using thermal evaporation in a vacuum chamber. Then, the Liq and Al were successively deposited at a rate of 0.1 and 5 Å s^−1^, respectively. The EL spectra, current efficiency (CE), power efficiency (PE), EQE, current–voltage–luminescence(C–V–L), and CIE of the OLEDs were recorded with an integrated optoelectronic performance test system with a calibrated spectra radiometer (TOPCON SR-UL1R) (Topcon Engineering Co., Ahmedabad, India) and Keithley 2400 source meter (Tektronix Co., Beaverton, OR, USA). All the measurements were conducted in a nitrogen-filled glove box at room temperature.

### 2.6. Calculation

The ground and excited states, adopting density functional theory (DFT) and time-dependent DFT (TD-DFT), were performed with Gaussian 09 software (ver. D.01, CT, USA) [17]; the cube files for the independent gradient model based on the Hirschfeld partition (IGMH), HOMO and the lowest unoccupied molecular ortibal (LUMO), and electron/hole distributions were generated from Multiwfn [18], and graphically visualized via Visual Molecular Dynamics (VMD, ver 1.9.4a, Champaign, IL, USA) [19]. The PBE/6–311(g) level for considering dispersion corrections was adopted for both ground and excited states. The molecular configuration for the ground-state calculation was taken from a crystalline structure, and vertical excited states were adopted for the calculations thereafter.

## 3. Results and Discussions

### 3.1. Single Crystal X-ray Diffraction (SC-XRD)

**Figure 1:**
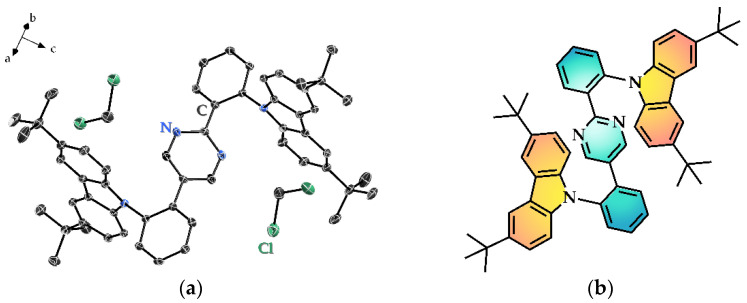
(**a**) The asymmetric unit of the crystalline structure of **PDPC**. Hydrogen atoms are omitted for clarity; (**b**) the chemical structure of **PDPC**, in which the acceptor part is tinted green, while the donor counterpart orange.

The compound **PDPC** crystallized in a *P*2_1_/*c* space group, as depicted in Figure 1. The general information and bond parameters for the refinement results of the crystal diffraction were tabled in Table S1 to S3. The asymmetric unit consisted of a whole target molecule with two dichloromethane solvent molecules. While refining the structure, the residual densities on the central pyrimidine ring remained unbalanced wherever the nitrogen atoms were denoted on either side (see Figure S5a,b in Supporting Information). Given the approximate atomic numbers of C and N, which may result in a similar diffraction intensity together with the symmetric skeleton of **PDPC**, we postulate that both orientations—either in the form displayed in Figure S5a or Figure S5b—evenly distributed in the crystal structure (Figure S5c). The fluctuation of density residues vanished on the pyrimidine ring after the operation, supporting the hypothesis that the two positions may coexist in the crystal.

**Figure 2:**
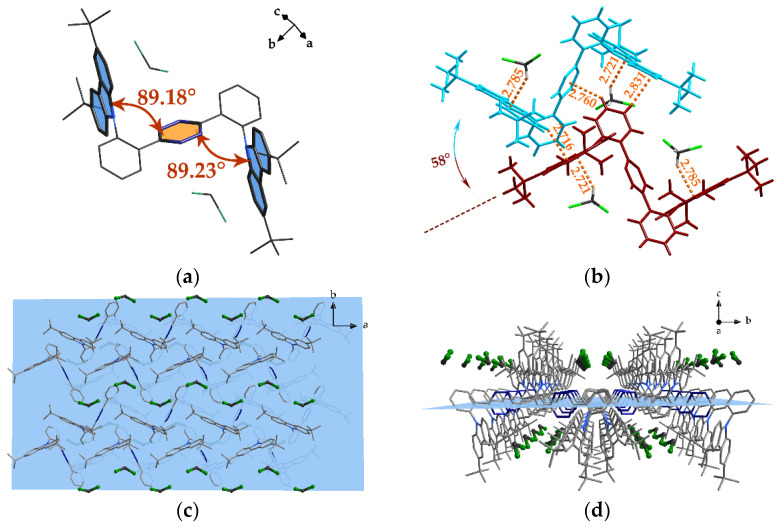
(**a**) The dihedral angle between the central pyrimidine plane and carbazole plane in **PDPC**; (**b**) intermolecular interactions between adjacent molecules and their surrounding solvent molecules, in which the **PDPC** molecules are colored as red and cyan; (**c**,**d**) the **PDPC** molecules packed in space, viewed in the *c* and *a* directions. The blue plane indicates the horizon where the central pyrimidine rings stand. The hydrogen atoms in subfigures (**a**,**c**,**d**) were omitted for clarity.

Owing to the steric hinderance of the ortho-position between the carbazole derivative group and central heterocyclic ring, the configuration of the molecule in the crystal folded to form an N-shape, in which the dihedral angle bended over 89° (Figure 2a). Another mirror-inverted molecule was stacked on the former one with a rotation angle of ca. 58° between the two carbazole planes of different molecules, capping on one of the joint phenyl rings of the first molecule, which connected the bis(tert-butyl)carbazole group with the central pyrimidine ring. Abundant intermolecular interactions, such as a substantial C-H…π secondary bond, were also found in this stacking configuration, the distance of which was measured to be between 2.72–2.83 Å (Figure 2b). A macroscopic view of the crystal revealed zig-zag tunnels walled by carbazole units. These tunnels were filled with solvent CH_2_Cl_2_ molecules, as illustrated in Figure 2c,d. To obtain a clear vision, a translucent blue plane was inserted where the central pyrimidine rings lie. Furthermore, the steric tert-butyl groups on carbazole rendered extra stability to the tunneling structure.

### 3.2. UV Diffuse Reflection Analysis

The UV–Vis diffuse reflection spectrum was obtained using the crystalline powder of **PDPC**. As shown in Figure S6a, the absorbance of the sample revealed the bandgap of the sample to be 2.64 eV, which agrees with the photoluminescence spectra illustrated in the following section. The high energy level of the bandgap can be attributed to the twisted structure of the molecule that prevented planar components in the molecule from further conjugation. 

### 3.3. Thermogravimetric Analysis (TGA)

The TGA curve is exhibited in Figure S6b. The slight decrease in weight before 200 °C could be attributed to the evaporation of the residue solvent molecules, while the sharp drop between 400 and 500 °C indicated the decomposition of the compound where the decomposition temperature was estimated to be around 450 °C.

### 3.4. Photoluminescent Properties

**Figure 3:**
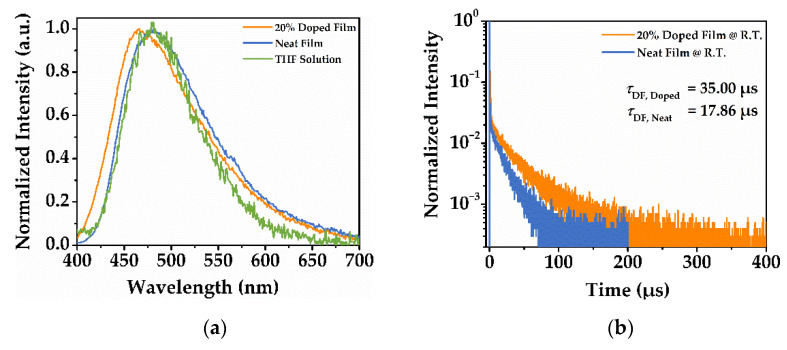
(**a**) The photoluminescence spectra; (**b**) the transient decay curve of **PDPC** film.

To investigate the excited-state properties of the material, the photoluminescent and electroluminescent properties of **PDPC** were measured. Figure 3 shows the photophysical properties of **PDPC** in the neat film and the 20% doping in BCPO (the structure is shown in Figure S11). The spectra of **PDPC** in a tetrahydrofuran solution were tested as a comparison. The photoluminescent spectra exhibited a nearly identical peak between the neat film and solution samples. However, a blue shift of around 15 nm of the emission peak was observed in the doped film sample. This was likely due to the dispersion of the **PDPC** molecules, which decreased the stacking and interactions between the **PDPC** molecules. The transient decay spectra of the films revealed the long lifetime of the photoluminescence, leading to 35.00 μs in doped film and 17.86 μs in neat film. Moreover, the lifetime of the neat film nearly halved compared with that of the doped film, indicating the concentration-induced quenching of the nondoped film. The time-resolved spectra of neat film was proven to have a thermally activated delayed fluorescent (TADF) characteristic, in which the spectra monitored during the first 10 ns remained unchanged after 100 μs. The spectra are depicted in Figure S7.

The photophysical parameters were quantified, and the results are collected in Table 1 to provide further insights. The parameters were calculated based on the previous report [20]. According to the results, the PLQY of the doped film surpassed that of the neat film by a small margin. The mediocre quantum yield could be explained by the competitive nonradiative transition that suppressed the transition rate of the excitons. The inspection of the decay rate for the films unveiled the faster prompt fluorescence of the doped film, which may have resulted in a higher-efficiency S_1_ → S_0_ emission. In addition, the long-lived delayed fluorescence and small *k*_RISC_ for both films lea to the conclusion that the TADF properties may not be distinct.

We also tested the luminescence and life of crystalline powder **PDPC**, as shown in Figure S9. The product exhibited blue emission with a maximum of around 460–480 nm under an excitation of 380 nm. The peak was fitted and four Gaussian bands were found, the peaks of which were located at 461, 483, 505, and 524 nm. The correspondent transient decay curves (Figure S9b) of these subpeaks were almost identical, suggesting these emissions may have stemmed from different vibrational levels of the same excited state. The fitting results for each decay curve are presented in Table S4.

### 3.5. Theoretical Calculation

**Figure 4:**
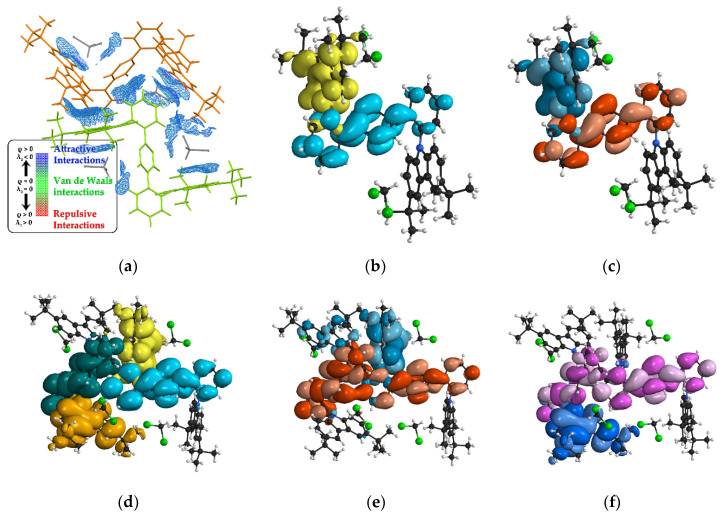
(**a**) The IGMH interactions between two neighboring **PDPC** molecules. The distribution of (**b**) holes (yellow) and electrons (cyan), and (**c**) HOMO (turquoise)/LUMO (red) orbitals in monomer **PDPC**; (**d**) the distribution of holes and electrons in dimer **PDPC**, under excited state S_1_ (hole: yellow; electron: cyan), and its degenerated state S_2_ (hole: orange; electron: green). The frontier orbitals of the dimer: (**e**) HOMO (turquoise)/LUMO (red) and (**f**) HOMO-1 (purple)/LUMO+1 (blue).

To theoretically investigate the interactions between the adjacent molecules, the independent gradient model based on the Hirschfeld partition (IGMH) method was adopted, and the results are visualized in Figure 4a [21]. The region filled with meshes indicated that there were interactions between molecules on both sides. The individual molecules are identically colored for clarity. According to the results, most of the interactions occurring between the molecules were attractive interactions, which are facilitative in forming multimers.

The calculation for a separated molecule revealed a charge-transfer (CT) state for the S_1_ state, in which the transition was mostly owing to the transfer from HOMO to LUMO (Figure 4b,c). When observing the dimeric configuration, we found a degenerated S_2_ state, in which the energy levels of S_1_ and S_2_ in the dimer were slightly lower than that in the monomer, while the oscillator strengths of the two degenerated S states almost remained as in the single molecule (Figure 4d). A further investigation into the frontier orbitals of the dimers unveiled that the distributions of LUMO and LUMO+1, the orbitals that substantially contribute to S_1_ and S_2_, resided on the acceptor segments of both molecules and conjugated as a whole, supporting the postulation that multimers were formed (Figure 4e,f). The information on the specific values are detailed in Table S5.

### 3.6. Electroluminescent Properties

**Figure 5:**
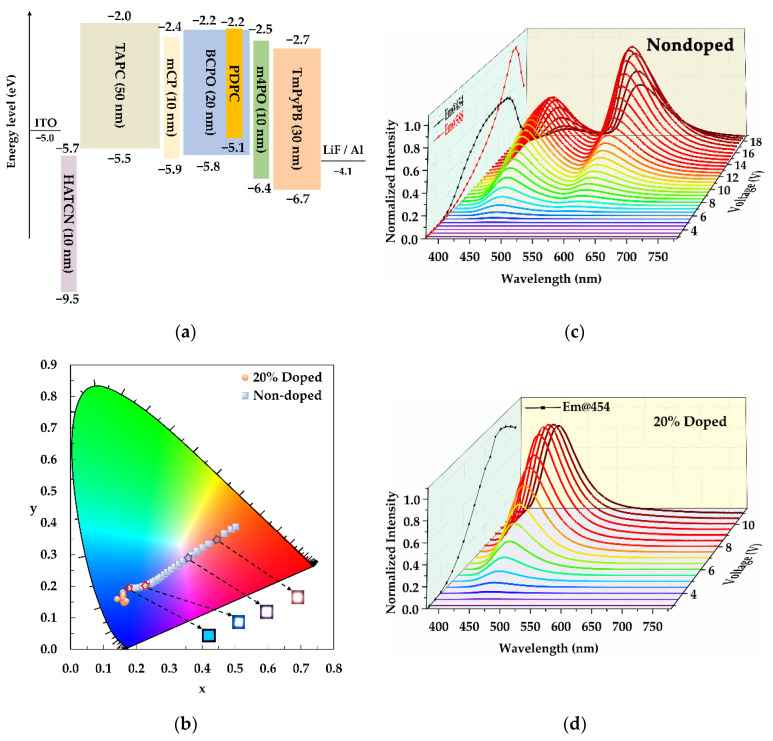
(**a**) Diagram for each layer in the fabrication of devices; (**b**) the coordinate plots of the nondoped and 20% doped devices on CIE 1931 color space; The electroluminescent curves of fabricated devices with an emitting layer for (**c**) nondoped and (**d**) 20% doped **PDPC**, under various voltages.

In order to examine the electroluminescent (EL) performance of the emitters, we fabricated nondoped and 20 wt%-doped OLEDs, the configurations of which [22,23] are depicted in Figure 5a. The energy level of **PDPC** was determined by CV analysis, the result of which is plotted in Figure S10b, and the bandgap was calculated from a Tauc plot that is shown in Figure S10c. The chemical structures of the used materials are drawn in Figure S11. The emission colors of the nondoped **PDPC** device drastically varied with increasing voltage, from blue, through pinkish white, to orange–red (Figure 5b). The EL spectra revealed two distinct bands peaking at around 460 and 590 nm (Figure 5c). The scatter-line figure referring to the maximum values of these peaks were projected on the left side wall of the 3D graph. When the voltage increased from 4 to 18 V, the intensities of both emission bands gradually increased, with varied intensity ratios. For instance, the blue-emission band dominated from 3 to 12 V, while the orange–red emission band was strengthened to be the predominant component at higher voltages (15–18 V). When the intensity of the latter emission peak surpassed its blue counterpart at 14.5 V, the device emitted a nearly white spectrum with a CIE 1931 coordinate of (0.35, 0.29) and a color rendering index (CRI) value of up to 93. As a comparative study, a device with an emitting layer of 20% **PDPC,** doped in BCPO, was fabricated, and the results are presented in Figure 5d. In contrast to the nondoped device, the doped device emitted blue light with indiscernible color change over a wide range of operating voltages. The EL spectra of the doped devices were voltage-independent and contained only one blue-emission band corresponding to the blue-emission band observed in the EL spectra of the nondoped device. The enlarged photos are displayed in Figure S12, and the parameters are listed in Table S6.

**Figure 6:**
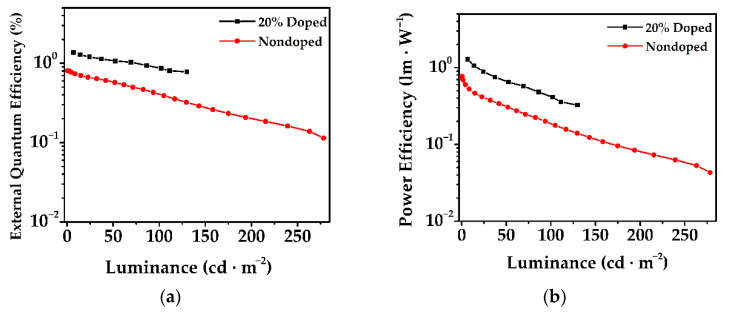

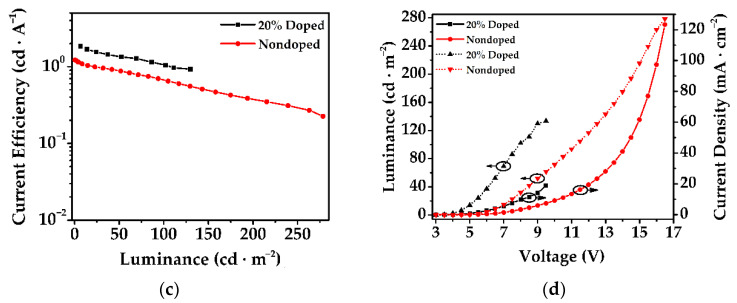
(**a**–**c**) The EQE, PE, and CE versus luminance plots, and (**d**) current density–luminance–voltage (J–L–V) plots for nondoped and doped devices. Circles and arrows in (**d**) indicated which side of the *y* axis the curve followed.

The key parameters for the electroluminescent performance of the doped and nondoped **PDPC** are compared in Figure 6. The lower turn-on voltage of the doped device (3.62 V) than that of the nondoped (5.19 V) indicated the efficient hole and electron injection of the former. In addition, the maximum EQE, PE, and CE for the doped device outperformed those of the nondoped device, which we attributed to the efficient separation of the excitons in doped devices, stemming from the dilution of **PDPC** that inhibited the concentration quenching. However, the maximum luminance of the nondoped device was over twice as much as that of the doped device. Based on the above-mentioned EL curves, it seemed plausible that the emission from the red region contributed to the high intensity of the luminance. However, both devices suffered from apparent efficiency roll-off. This could have been the result of the long-lived delayed fluorescence lifetimes (17.86 and 35.00 μs) and slow RISC rates (2.86 × 10^4^ and 5.60 × 10^4^ s^−1^).

**Figure 7:**
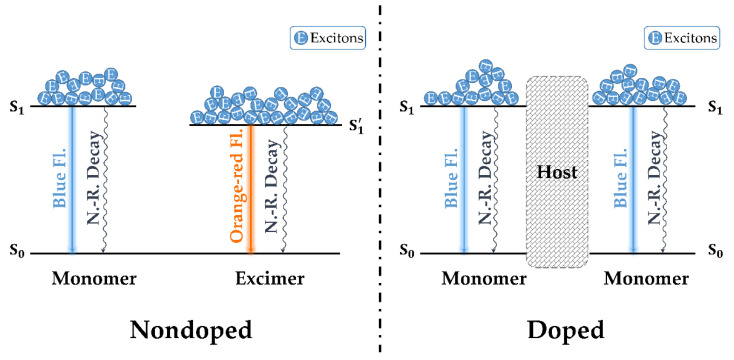
A proposed mechanism illustrating the discrepancies of electroluminescence between nondoped and doped devices.

Thus, the orange–red emission band appearing only in the nondoped device could be attributed to the emission of excimers, as illustrated in Figure 7 [24,25,26]. In the nondoped device, the **PDPC** molecules were highly aggregated, leading to the possibility of forming excimers with a stabilized emissive state at a high-enough voltage. With an increase in voltage, the excitons’ recombined emission on the excimers sharply increased, resulting in a gradually redshifted emission color. The nondoped device failed when the voltage was higher than 16.5 V. However, when the emitter molecules were well-dispersed into the host BCPO, the formation of excimers was completely suppressed, with the only emission coming from the monomers.

## 4. Conclusions

We designed and synthesized an organic molecule featuring a D–A–D structure with two ortho-substituted 3,6-di-tert-butyl-9*H*-carbazoles as donors and a central pyrimidine as the acceptor. The compound exhibited blue emission peaking at 454 nm under UV light. Using this compound as a single emitter, the nondoped OLED showed a unique voltage-dependent EL with emission colors spanning from blue to orange–red. The EL color of the nondoped device varied from blue to orange–red with an increase in voltage, during which a pinkish-white light with a CIE coordinate of (0.35, 0.29) and a CRI value of 93 was observed. We inferred from experimental and theoretical investigations that the electro-induced orangish-red emission stemmed from the excitation of multimers, which were formed via intermolecular interactions. Despite the TADF characteristics of the compound, the long-lived decay, small *k*_RISC_, and competitive *k*_nr_ against *k*_r_ altogether lead to the concentration quenching of the exciton, which may be attributed to the depressed EL efficiencies. This study provides insight into the multimer-excited mechanism and may help to develop novel color-tunable OLEDs.

## Figures and Tables

**Table 1:** Photophysical parameters of **PDPC** in 20% doped and neat films.

Film	*λ*_PL_ ^(a)^ (nm)	*Φ*_PL_^(b)^ (%)	*τ*_PF_/*τ*_DF_^(c)^ (ns/μs)	*k*_PF_ ^(d)^ (10^8^ s^−1^)	*k*_DF_ ^(e)^ (10^4^ s^−1^)	*k*_r_^S^/ *k_n_*_r_^S (f)^ (10^8^ s^−1^)	*k*_RISC_^(g)^ (10^4^ s^−1^)
Doped	466	52	1.12 ^(h)^/35.00	8.93	2.86	4.64/4.29	2.86
Neat	484	46	2.23 ^(h)^/17.86	4.48	5.60	2.06/2.42	5.60

^(a)^ Maximum of the photoluminescent spectrum; ^(b)^ photoluminescence quantum yield (PLQY); ^(c)^ prompt and delayed fluorescence; ^(d)^ rate constant of prompt fluorescence; ^(e)^ rate constant of delayed fluorescence; ^(f)^ rate constant of radiative and non-radiative transitions for S_1_ state; ^(g)^ rate constant of the reverse intersystem crossing between S_1_ and T_1_ state; ^(h)^ transient decay spectra were plotted in Figure S8.

## Data Availability

The crystal data can be found at The Cambridge Crystallographic Data Centre (www.ccdc.cam.ac.uk (accessed on 8 May 2022)), No. 2171367.

## Supplementary Materials

The following supporting information can be downloaded at: https://www.mdpi.com/article/10.3390/nano12142333/s1, Scheme S1: Synthetic route of 2,5-bis(2-fluorophenyl)pyrimidine; Scheme S2: Synthetic route of 9-(2,3-bis((2-bromophenyl)thio)phenyl)-9*H*-carbazole (**PDPC**); Figure S1: ^1^H NMR spectrum of 2,5-bis(2-fluorophenyl)pyrimidine; Figure S2: ^13^C NMR spectrum of 2,5-bis(2-fluorophenyl)pyrimidine; Figure S3: ^1^H NMR spectrum of **PDPC**; Figure S4: 13C NMR spectrum of **PDPC**; Figure S5: Density residue for different denotations of the pyrimidine ring; Table S1: Single-crystal data for **PDPC**; Table S2: Selected bond lengths for **PDPC**; Table S3: Selected bond angles for **PDPC**; Figure S6: (a) UV-vis absorption spectrum of solid state **PDPC**; (b) TGA curve of **PDPC**; Figure S7: The normalized time-resolved photoluminescent spectra of neat-film **PDPC** within different time ranges under ambient temperature; Figure S8: The transient decay spectra of doped- and neat-film **PDPC** at room temperature; Figure S9: The (a) photoluminescence spectra and (b) transient decay curve of **PDPC** powder; Table S4: Photoluminescent lifetimes and percentages (A) of crystalline **PDPC**; Table S5: Component analysis of the excited-state **PDPC** monomers and dimers; Figure S10: The cyclic voltammetric plots for (a) ferrocene and (b) **PDPC**; (c) Tauc plot for UV-vis absorption of **PDPC** solution (ca. 10^−5^ mol/L); Figure S11: Chemical formula for the abbreviations in the fabricated device; Figure S12: Enlarged photo shots for the nondoped device; Table S6: Parameters for the nondoped device. Reference [27] are cited in the supplementary materials.
