# Supplementary material for: Voltage-Dependent Emission Varying from Blue to Orange–Red from a Nondoped Organic Light-Emitting Diode with a Single Emitter
Source: Nanomaterials (Basel). 2022 Jul 7;12(14):2333. doi: 10.3390/nano12142333 (PMC9320025; doi:10.3390/nano12142333)
Supplement: Supplementary file 1 [file nanomaterials-12-02333-s001.zip › nanomaterials-1783900-supplementary.pdf]

nanomaterials

Supporting information

Voltage-Dependent Emission Varying from Blue to Orange–Red from a Nondoped Organic Light-Emitting Diode with a Single Emitter

Mingxue Yang [1,2], Tian-Xiang Zhao [1,2], Si-Chao Ji [1,2], Xiao-Dong Tao [1,2], Xu-Lin Chen [1,2], Lingyi Meng [1,2], Dong Liang [1] and Can-Zhong Lu [1,2,3,*]

[1] CAS Key Laboratory of Design and Assembly of Functional Nanostructures, and Fujian Provincial Key Laboratory of Nanomaterials, Fujian Institute of Research on the Structure of Matter, Chinese Academy of Sciences, Fuzhou 350002, China; xmyangmingxue@fjirsm.ac.cn (M.Y.); xmzhaotianxiang@fjirsm.ac.cn (T.-X.Z.); xmjisichao@fjirsm.ac.cn (S.-C.J.); taoxiaodong17@mails.ucas.edu.cn (X.-D.T.); xlchem@fjirsm.ac.cn (X.-L.C.); lymeng@fjirsm.ac.cn (L.M.); dl@fjirsm.ac.cn (D.L.)

[2] Xiamen Institute of Rare Earth Materials, Fujian Institute of Research on the Structure of Matter, Chinese Academy of Sciences, Xiamen 361021, China

[3] University of Chinese Academy of Sciences, Beijing 100049, China

* Correspondence: czlu@fjirsm.ac.cn

1. Synthetic schemes of Materials

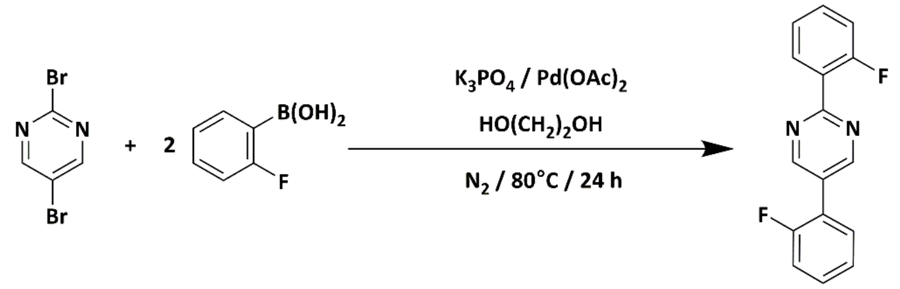

Scheme S1 Synthetic route of 2,5-bis(2-fluorophenyl)pyrimidine

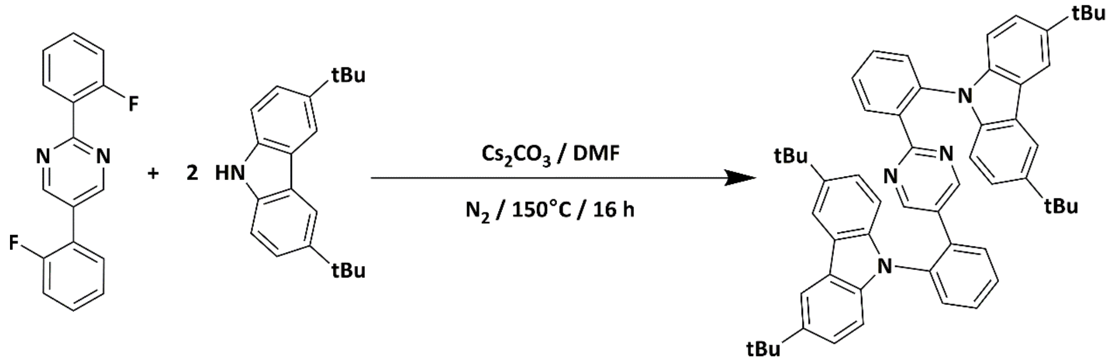

Scheme S2 Synthetic route of 9-(2,3-bis((2-bromophenyl)thio)phenyl)-9*H*-carbazole (**PDPC**)

2. NMR Spectra

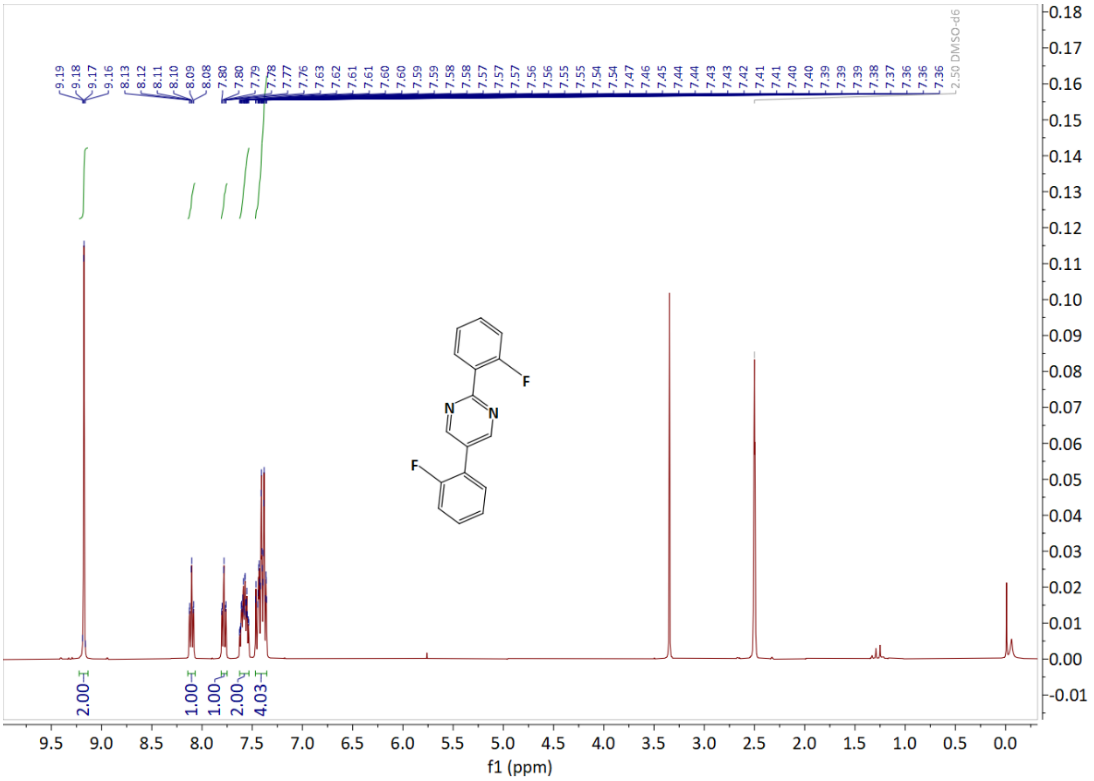

Figure S1 ^{1}H NMR spectrum of 2,5-bis(2-fluorophenyl)pyrimidine

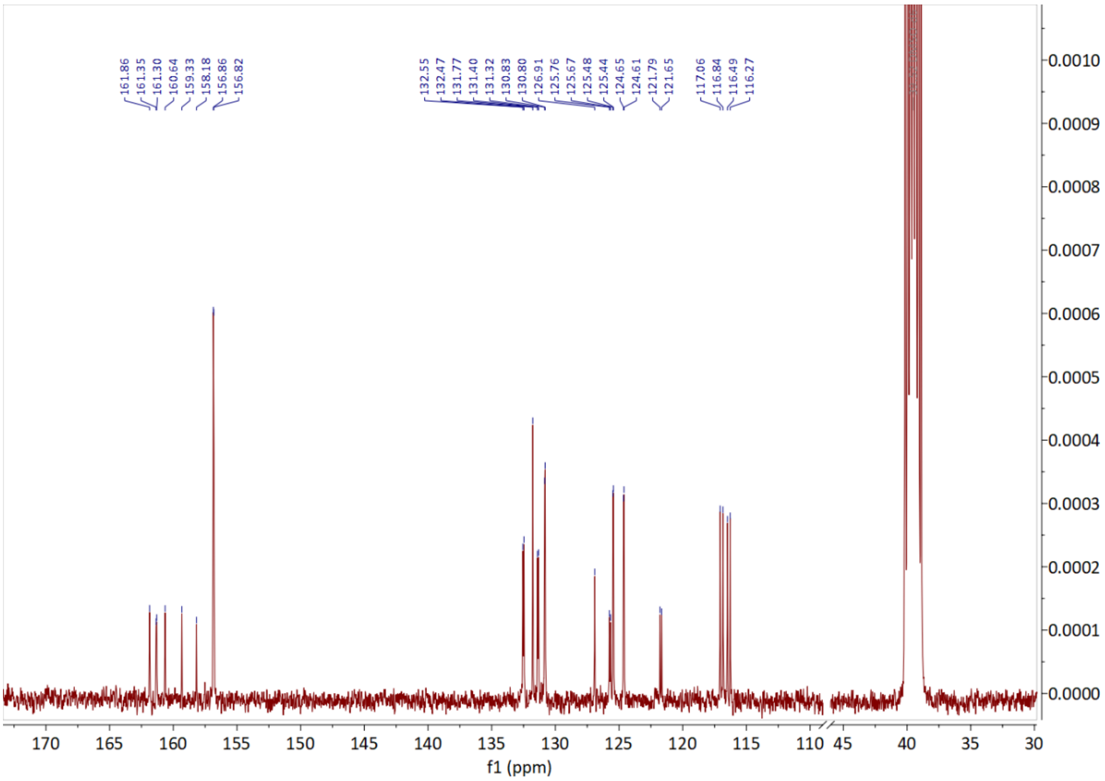

Figure S2 ^{13}C NMR spectrum of 2,5-bis(2-fluorophenyl)pyrimidine

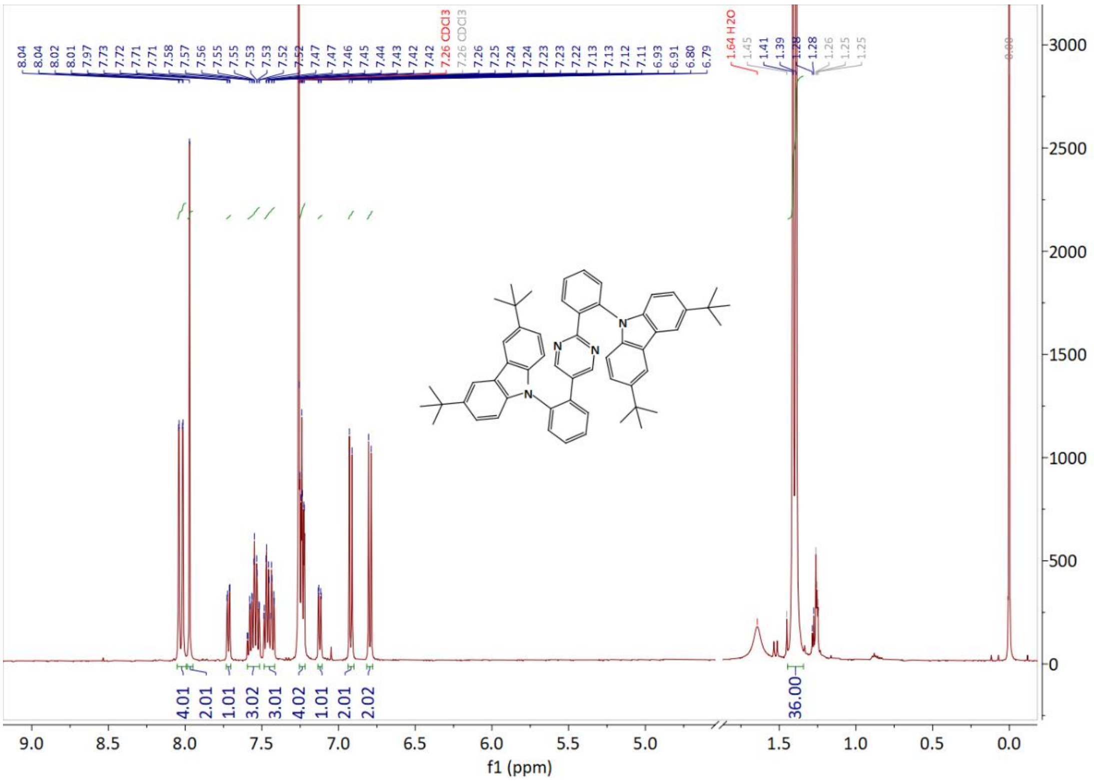

Figure S3 ^{1}H NMR spectrum of **PDPC**

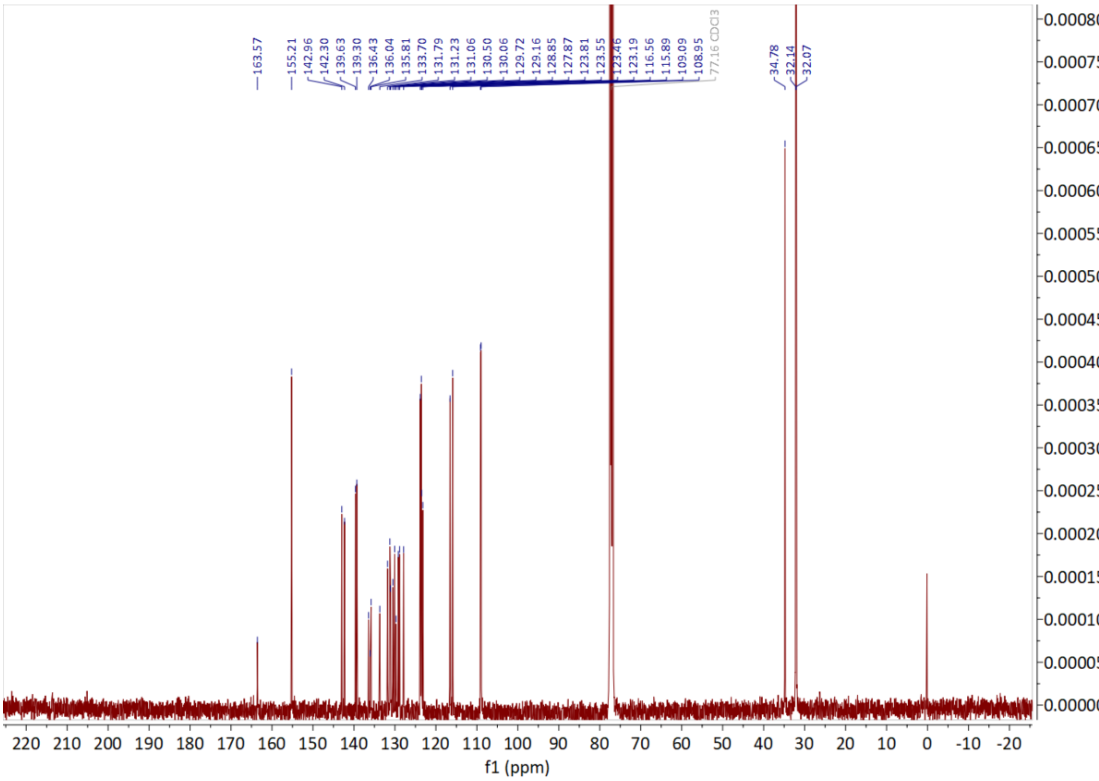

Figure S4 ^{13}C NMR spectrum of **PDPC**

3. Single Crystals Analysis

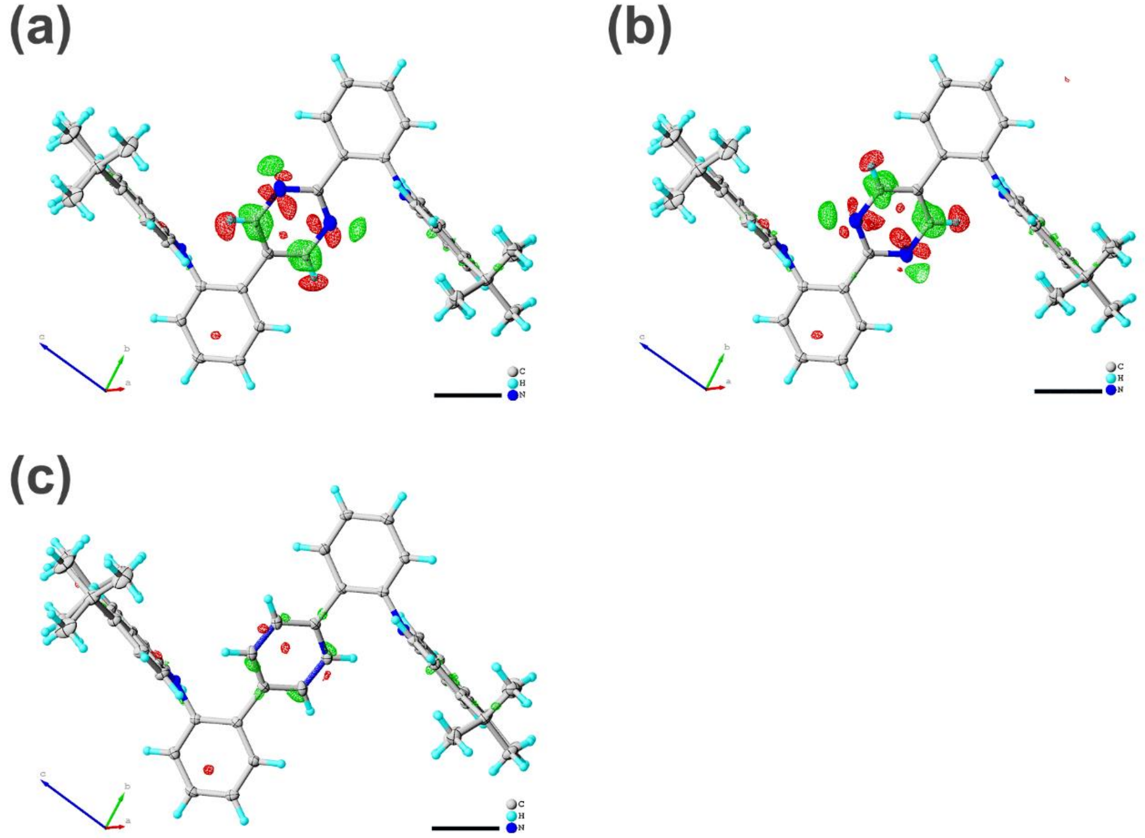

Figure S5 Density residue for different denotations of pyrimidine ring, in which the wires in green color indicate positive and red negative. The N and C atoms are individual as for **(a)** and **(b)**, while 50% of C / N atoms are denoted in **(c)**

Table S1 Single crystals data for **PDPC**

Item	Value
Chemical formula	$C_{56}H_{58}N_4 \cdot 2(CH_2Cl_2)$
Formula weight	956.91
Temperature / K	100.01(15)
Crystal system	monoclinic
Space group	*P*21/*c*
a / Å	11.3239(4)
b / Å	13.6212(4)
c / Å	32.8757(11)
α / °	90
β / °	90.735(3)
γ / °	90
Volume / $Å^3$	5070.5(3)
Z	4
ρ_{calc} / $g \cdot cm^{-3}$	1.254
μ / mm^{-1}	2.438
F(000)	2024.0
Crystal size / mm^3	0.15 × 0.18 × 0.2
Radiation	Cu Kα (λ = 1.54184)
2Θ range for data collection / °	7.024 to 146.95
Index ranges	−11≤h≤14, −16≤k≤16, −40≤l≤32
Reflections collected	24234
Independent reflections	9961 [R_{int} = 0.0302, R_{σ} = 0.0369]
Data / restraints / parameters	9961/10/608
Goodness-of-fit on F^2	1.025
Final R indexes [I>=2σ (I)]	R1 = 0.0452, wR2 = 0.1164
Final R indexes [all data]	R1 = 0.0642, wR2 = 0.1302
Largest diff. peak/hole / $e \cdot Å^{-3}$	0.51/−0.59

Table S2 Selected bond lengths for **PDPC**

Atom	Atom	Length / Å	Atom	Atom	Length / Å	Atom	Atom	Length / Å
N3A	C11A	1.398(2)	C3	N2	1.364(2)	C1	N2A	1.367(2)
N3A	C10A	1.430(2)	C3	N4	1.371(2)	C17	C22	1.405(2)
N3A	C22A	1.399(2)	C3	C2A	1.364(2)	C17	C18	1.403(2)
N3	C11	1.394(2)	C3	C4A	1.371(2)	C13	C12	1.392(2)
N3	C10	1.431(2)	C11A	C16A	1.405(3)	C13	C14	1.412(3)
N3	C22	1.399(2)	C11A	C12A	1.396(2)	C15	C14	1.393(2)
C11	C16	1.412(2)	C10A	C5A	1.406(2)	C16A	C17A	1.450(2)
C11	C12	1.394(2)	C10A	C9A	1.392(2)	C16A	C15A	1.401(3)

Atom	Atom	Length / Å	Atom	Atom	Length / Å	Atom	Atom	Length / Å
C16	C17	1.448(2)	C5A	C1	1.490(2)	C22	C21	1.396(2)
C16	C15	1.397(2)	C5A	C6A	1.398(3)	C14	C23	1.534(2)
C10	C9	1.392(2)	C1	C2	1.367(2)	C18	C19	1.391(3)
C10	C5	1.402(2)	C1	C4	1.375(2)	C12A	C13A	1.390(3)
C3	C5	1.491(2)	C1	N4A	1.375(2)	C9	C8	1.390(3)

Table S3 Selected bond angles for **PDPC**

Atom	Atom	Atom	Angle/°	Atom	Atom	Atom	Angle/°
C11A	N3A	C10A	125.02(14)	C21A	C22A	N3A	129.33(16)
C11A	N3A	C22A	107.78(14)	C21A	C22A	C17A	121.22(16)
C22A	N3A	C10A	124.39(14)	C7A	C6A	C5A	121.72(17)
C11	N3	C10	124.96(14)	C15A	C14A	C13A	118.20(16)
C11	N3	C22	108.02(14)	C15A	C14A	C23A	122.06(16)
C22	N3	C10	124.36(14)	C13A	C14A	C23A	119.68(15)
N3	C11	C16	109.11(15)	C14A	C15A	C16A	120.03(17)
N3	C11	C12	129.90(16)	C20A	C21A	C22A	117.48(17)
C12	C11	C16	120.98(16)	C7	C6	C5	121.47(17)
C11	C16	C17	106.73(15)	C21	C20	C19	123.10(17)
C15	C16	C11	120.13(16)	C20A	C19A	C27A	118.83(17)
C15	C16	C17	133.09(16)	C18A	C19A	C20A	118.09(17)
C9	C10	N3	117.90(16)	C18A	C19A	C27A	123.06(17)
C9	C10	C5	119.98(16)	C21A	C20A	C19A	123.24(17)
C5	C10	N3	122.12(16)	C29	C27	C30	108.56(18)
N2	C3	C5	122.29(16)	C28	C27	C29	108.12(19)
N2	C3	N4	120.40(16)	C28	C27	C30	109.21(19)
N4	C3	C5	117.32(16)	C19	C27	C29	111.90(16)
C2A	C3	C5	122.29(16)	C19	C27	C30	109.46(17)
C2A	C3	C4A	120.40(16)	C19	C27	C28	109.55(17)
C4A	C3	C5	117.32(16)	C19A	C27A	C28A	108.89(18)
N3A	C11A	C16A	109.12(15)	C29A	C27A	C19A	112.01(17)
C12A	C11A	N3A	129.67(17)	C29A	C27A	C28A	108.3(2)
C12A	C11A	C16A	121.20(16)	C29A	C27A	C30A	108.6(2)
C5A	C10A	N3A	121.99(15)	C30A	C27A	C19A	109.98(18)
C9A	C10A	N3A	117.94(16)	C30A	C27A	C28A	109.0(2)
C9A	C10A	C5A	120.07(16)	C6A	C7A	C8A	119.51(17)
C10A	C5A	C1	123.21(16)	C12A	C13A	C14A	123.16(16)
C6A	C5A	C10A	118.12(16)	C14	C23	C24	108.65(15)

4. UV-Vis Analysis and Thermogravimetry

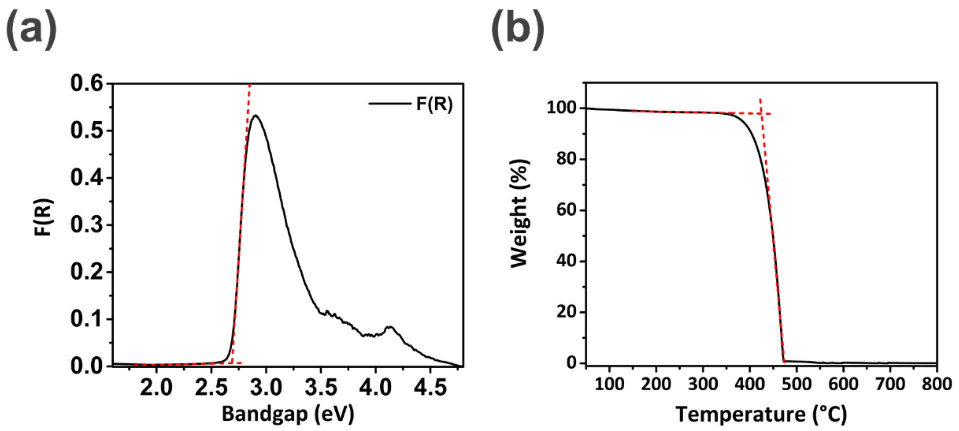

Figure S6 (a) UV-vis absorption spectrum of solid state **PDPC**; **(b)** TGA curve of **PDPC**

5. Photophysical Properties

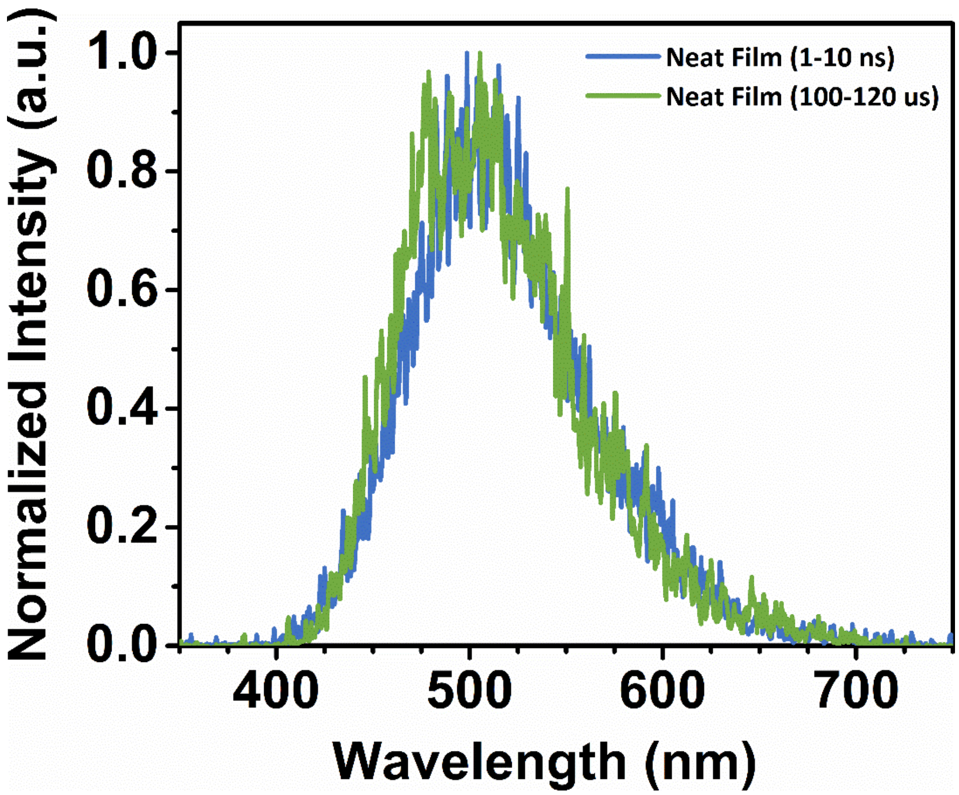

Figure S7 The normalized time-resolved photoluminescent spectra of neat film **PDPC** in different time ranges under ambient temperature

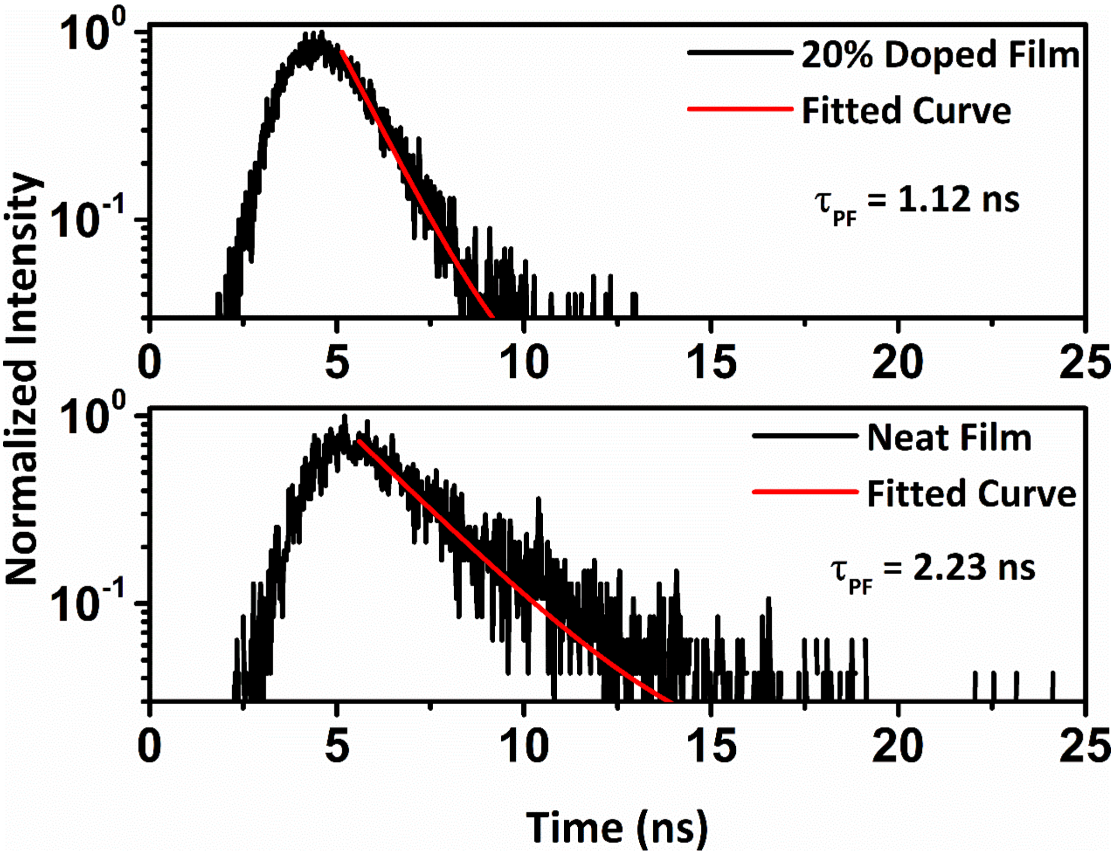

Figure S8 The transient decay spectra of doped and neat film **PDPC** at room temperature

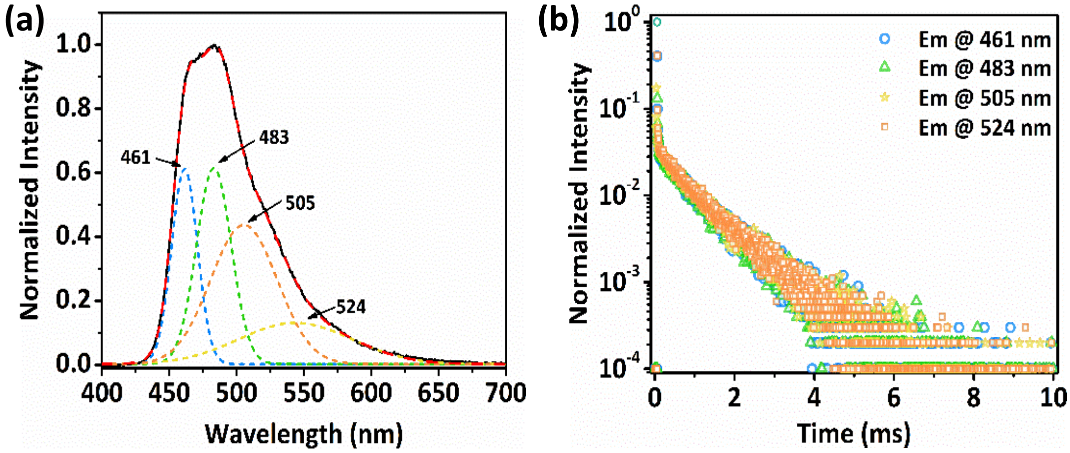

Figure S9 The **(a)** photoluminescence spectra and **(b)** transient decay curve of PDPC powder. The solid black line in **(a)** is the original spectrum, while the short-dashed lines in blue, green, orange and yellow represent the fitted gaussian peaks, and the sum of the fitted peaks was plotted in red dashed line. The colors in **(b)** corresponds to that in **(a)**. The peak fitting was proceeded through "Peak Analyzer" function in OriginPro software [1] ,and the fitted curves comply to "Area version of Gaussian Function":

$$y = y_0 + \frac{Ae^{\frac{-4\ln(2)(x-x_c)^2}{w^2}}}{w\sqrt{\frac{\pi}{4\ln(2)}}}$$

In which A, x_c and w indicated the area, center x value, and FWHM, respectively.

Table S4 Photoluminescent lifetimes and percentages (*A*) of crystalline **PDPC**

Wavelength(nm)	τ_1 (μs)	A_1 (%)	τ_2 (μs)	A_2 (%)	$\bar{\tau}$(μs)
461	188.87	8.11	995.16	91.89	929.76
483	130.24	5.34	968.94	94.66	924.15
524	179.16	8.17	1003.61	91.83	936.25

6. Theoretical Calculation

Table S5 Component analysis of the excited-state **PDPC** monomer and dimer

	**States**	**Energy (eV)**	**Transition Contributions [a] (%)**	**Oscillator Strength**
Monomer	S_1	3.2736	H→L(97.97%)	0.0164
	S_2	3.4785	H-1→L(97.72%)	0.0130
	S_3	3.6437	H→L+1(97.33%)	0.0241
	S_4	3.6856	H-2→L(94.48%)	0.0078
	S_5	3.7402	H-1→L+1(95.88%)	0.0291
	S_6	3.8607	H-3→L(96.05%)	0.0021
	S_7	3.9438	H-2→L+1(41.31%), H-7→L+1(16.95%), H-4→L+1(14.99%), H-7→L(4.97%), H-4→L(4.91%)	0.0053
	S_8	4.0128	H→L+3(82.95%), H-4→L(3.35%)	0.0472
	S_9	4.0273	H-4→L(26.28%), H-7→L(23.85%), H-2→L+1(22.53%), H→L+3(8.69%), H-5→L(5.72%), H-6→L(3.61%)	0.0106
	S_{10}	4.0530	H-1→L+2(83.95%), H-3→L+1(8.49%), H-3→L+7(3.06%)	0.0301
Dimer	S_1	3.2620	H→L+1(55.04%), H→L(38.49%)	0.0128
	S_1*	3.2693	H-1→L(65.25%), H-1→L+1(30.58%)	0.0165
	S_3	3.4084	H-2→L(53.11%), H-2→L+1(21.53%), H→L(13.77%), H→L+1(9.08%)	0.0084
	S_4	3.4753	H-3→L+1(59.25%), H-3→L(37.24%)	0.0135
	S_5	3.4873	H→L(43.32%), H→L+1(31.41%), H-2→L(15.39%), H-2→L+1(7.07%)	0.0024
	S_6	3.5470	H-1→L+1(58.28%), H-1→L(27.99%), H-1→L+2(10.77%)	0.0040
	S_7	3.6471	H-2→L+1(24.68%), H-2→L+2(21.24%), H-4→L+1(16.23%), H-4→L(12.13%), H-2→L(9.74%), H→L+2(3.07%)	0.0148
	S_8	3.6611	H→L+3(67.28%), H→L+2(10.84%), H-1→L+2(7.17%)	0.0047
	S_9	3.6662	H-1→L+2(56.73%), H→L+3(11.41%), H-5→L(11.02%), H-1→L+1(5.44%), H-5→L+1(4.56%), H-4→L+1(3.17%)	0.0231
	S_{10}	3.6738	H-5→L(41.06%), H-5→L+1(20.19%), H-4→L(12.11%), H→L+3(5.84%), H-2→L+2(5.39%), H-4→L+1(4.16%), H-1→L+2(3.99%)	0.0298

[a] H: HOMO; L: LUMO

* Degenerated orbital.

7. Electroluminescence Properties

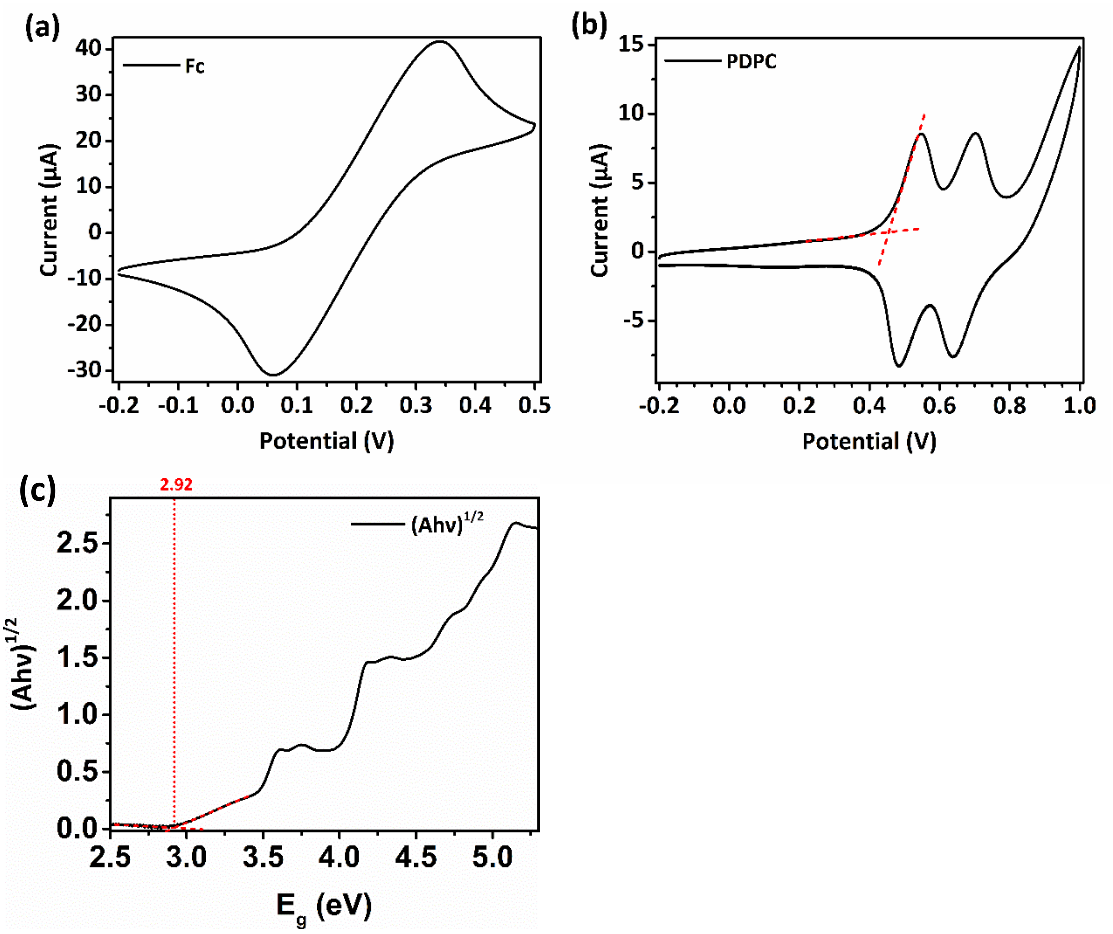

Figure S10 The cyclic voltametric plots for **(a)** ferrocene and **(b) PDPC**; **(c)** Tauc plot for UV-vis absorption of **PDPC** solution (c.a. 10^{-5} mol/L)

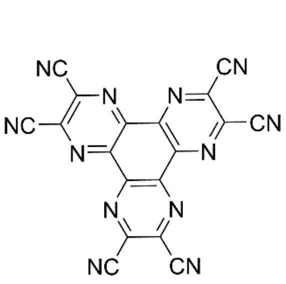

HATCN

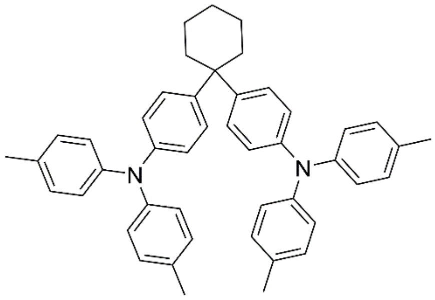

TAPC

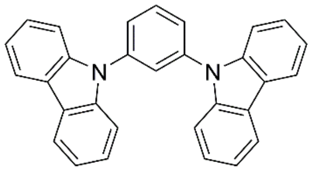

mCP

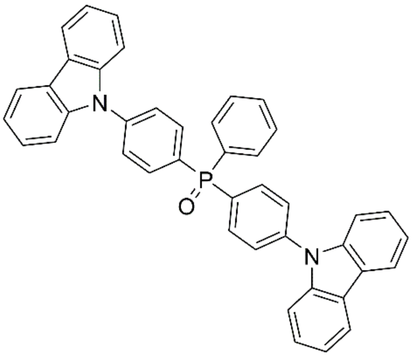

BCPO

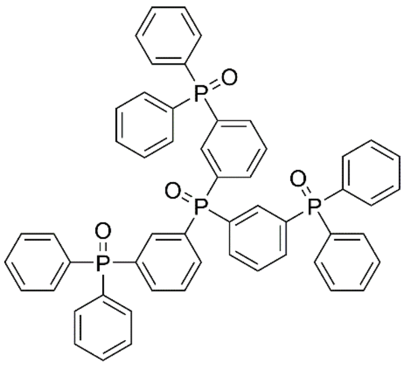

M4PO

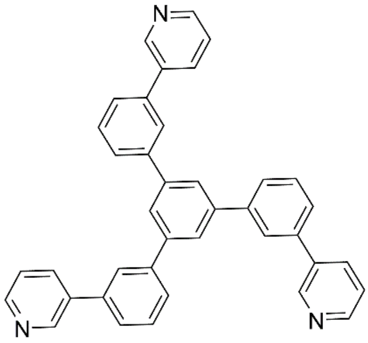

TmPyPB

Figure S11 Chemical formula to the abbreviations in the fabricated device

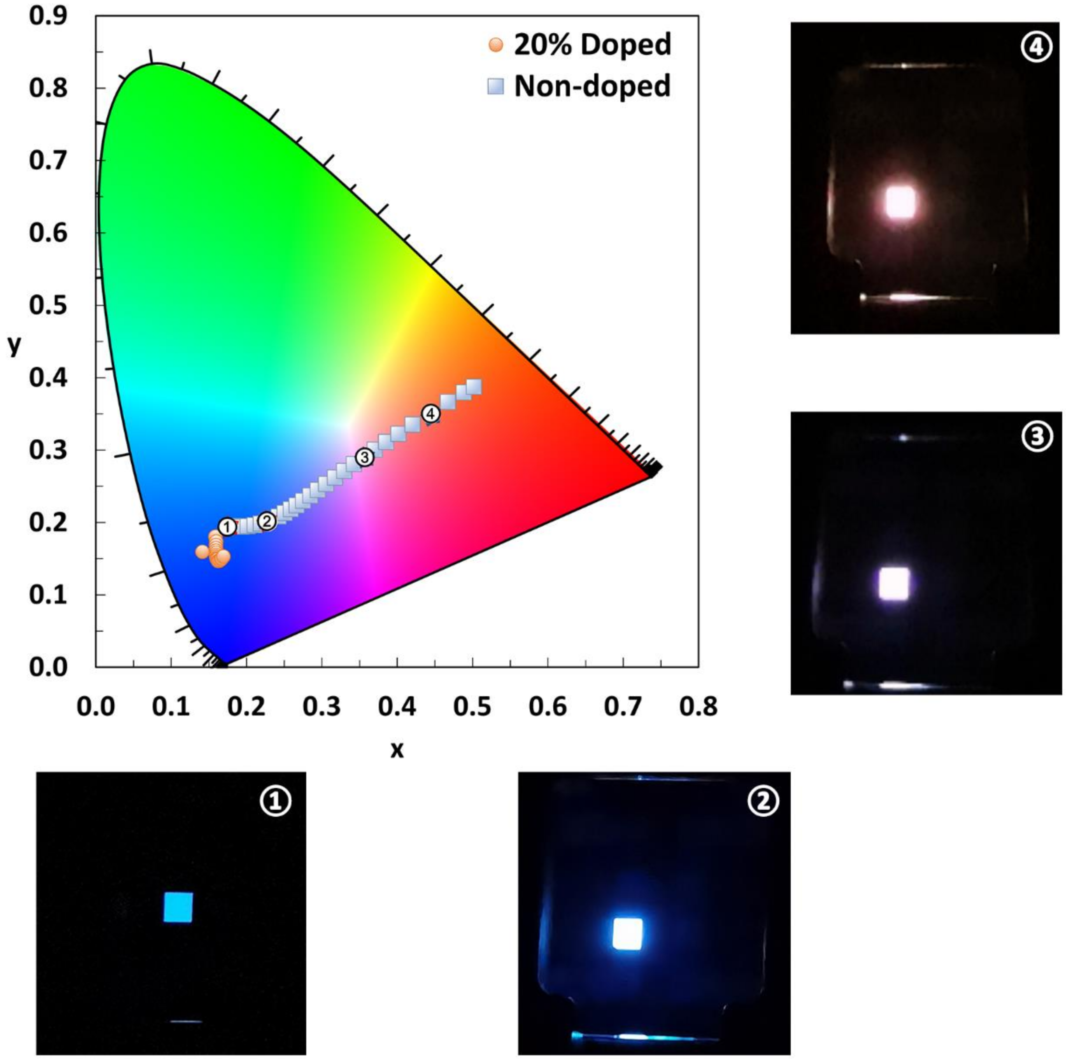

Figure S12 Enlarged photo shots for nondoped device

Table S6 Parameters for doped and nondoped device

Emitter	λ_{EL} a) (nm)	CIE b) (x, y)	CRI_{max} c)	L_{max} d) (cd / m^2)	CE_{max} e) (cd / A)	PE_{max} f) (lm / W)	EQE_{max} g)
Nondoped	454 / 588	(0.35, 0.29)	93	278.4	1.22	0.77	0.81
20% Doped	454	(0.16, 0.17)	–	133.6	2.16	1.94	1.53

a) Wavelength of emission peaks; b) CIE coordinate nearest to (0.33, 0.33); c) The optimum Color Rendering Index; d)–g) The maximum luminance, current efficiency, power efficiency and external quantum efficiency.

Reference

1. OriginPro, Learning Edition. OriginLab Corporation: Northampton, MA, USA. Available online: https://www.originlab.com/originprolearning.aspx (accessed on 6 June 2022).
